# Surface Modification of Bentonite with Polymer Brushes and Its Application as an Efficient Adsorbent for the Removal of Hazardous Dye Orange I

**DOI:** 10.3390/nano10061112

**Published:** 2020-06-04

**Authors:** Wenjuan Guo, Ahmad Umar, Yankai Du, Luyan Wang, Meishan Pei

**Affiliations:** 1 Institute of Surface Analysis and Chemical Biology, University of Jinan, Jinan 250022, China; 2 Department of Chemistry, Faculty of Science and Arts, Promising Centre for Sensors and Electronic Devices, Najran University, Najran 11001, Saudi Arabia; 3 School of Chemistry and Chemical Engineering, University of Jinan, Jinan 250022, China; yankaiwill@126.com (Y.D.); chm_wangly@ujn.edu.cn (L.W.)

**Keywords:** PDMAEMA, polymer brushes, Orange I, dyes removal, water treatment

## Abstract

Poly(2-(dimethylamino)ethyl methacrylate)-grafted bentonite, marked as Bent-PDMAEMA, was designed and prepared by a surface-initiated atom transfer radical polymerization method for the first time in this study. Fourier transform infrared spectroscopy (FTIR), X-ray diffraction (XRD), scanning electron microscopy (SEM) and thermal gravimetric analysis (TGA) were applied to characterize the structure of Bent-PDMAEMA, which resulted in the successful synthesis of Bent-PDMAEMA. As a cationic adsorbent, the designed Bent-PDMAEMA was used to remove dye Orange I from wastewater. The adsorption property of Bent-PDMAEMA for Orange I dye was investigated under different experimental conditions, such as solution pH, initial dye concentration, contact time and temperature. Under the optimum conditions, the adsorption amount of Bent-PDMAEMA for Orange I dye could reach 700 mg·g^−1^, indicating the potential application of Bent-PDMAEMA for anionic dyes in the treatment of wastewater. Moreover, the experimental data fitted well with the Langmuir model. The adsorption process obeyed pseudo-second-order kinetic process mechanism.

## 1. Introduction

Recently, the environmental pollution caused by colored wastewater has significantly increased due to the huge expansion of related industries, and threatened not only aquatic life but also that of human beings and animals [1,2,3]. Most of the colored wastewater containing dyes, employed as coloring agents, are being released from industrial fields such as leather, cosmetics, food and mineral processing, coatings, rubber and so on [4,5,6]. Among various different dyes, azo dyes are difficult to degrade, and thus severely contaminate water and threaten living beings [7]. Therefore, wastewater which contains azo dyes should be tackled before it discharges into flowing water such as rivers, lakes etc. There are various methods such as photolysis, adsorption, electro-Fenton process, photocatalytic process, bio-contact oxidation and so on, which are commonly used to purify wastewater containing highly harmful and toxic azo dyes [4,5,6,7,8,9,10,11,12,13,14,15]. Among several technologies, the adsorption process is a convenient, efficient and also the most versatile method [10,11,12,13,14,15]. Novel materials with a higher adsorption amount have been synthesized for the removal of dyes, such as ultrathin sodium ferric silicate 2D nanosheets [16], Bi_2_WO_6_/BiOCl hierarchical structure [17] and zero-valent iron [18]. Nanoparticles in particular have novel characteristics which enable them to be chosen as ideal adsorbents for efficiently decontaminating water. Almeida et al. investigated biological methods for removing azo dyes [8], which showed that the adsorption capacity of fungal biomass to dyes was excellent. Aliouche et al. found that color removal caused by azo dyes followed an increasing order: UV < acetone/UV < S_2_O_8_^2−^/heat < S_2_O_8_^2−^/H_2_O_2_/UV < S_2_O_8_^2−^/UV < H_2_O_2_/UV [9]. Various nanomaterials used as the absorbents contain cobalt-zinc ferrite nanoadsorbents [19], silica nano-composite [16,20,21,22], nano zero-valent iron [23], Fe_3_O_4_ nano-composite [24,25,26,27], carbon nanomaterials [28,29,30,31,32,33] and clay [34,35].

Due to it being naturally occurring and easily available, bentonite (Bent) has attracted significant interest as an adsorbent. Berez et al. reported the wastewater treatment by the physisorption of Foron Blue 291 on natural bentonite under both static and dynamic flow conditions of the aqueous solution [36], while the poor dispersibility of the natural Bent limited its adsorption property. In order to improve its dispersibility, some special functional groups should be modified onto a Bent surface to enhance the adsorption amount of the natural Bent. At the same time, the natural Bent possesses negative charges, which limits its application in the removal of anionic dyes. Therefore, the natural Bent should be modified with organics by the chemical reaction of hydroxyl groups of Bent, or by the direct exchange of organic cations into the interlayer of the natural Bent. Polyaniline-intercalated Bent has been proven to be an efficient adsorbent for Reactive Red 2 [37]. Its adsorption amount could reach 257.7 mg/g. The designed organophilic Bent has the potential properties for the adsorption of organic dyes with anionic groups.

In order to further improve the adsorption amount, the adsorbent can usually be modified with much more functional groups to form polymer brushes [38,39,40]. Among the grafting methods, the surface-initiated atom transfers radical polymerization (SI-ATRP) technique is a ‘living’/controlled polymerization method which can provide much denser polymer brushes on the substrates. Polymers obtained by SI-ATRP have many excellent properties, such as controlled molecular weight, molecular weight distribution and a well-defined structure. In the SI-ATRP procedures, almost all the polymers are connected to the Bent by chemical bonds.

Here, poly(2-(dimethylamino) ethyl methacrylate) (PDMAEMA), which has an amino group in the repeat unit, was designed to be grafted on Bent. The amino groups of PDMAEMA brushes provide the adsorption sites. All the products in every polymerization process were characterized. The as-prepared material was used to adsorb Orange I (OI) with negative charges. The effects of Orange I initial concentration, solution pH, contact time and environmental temperature on the adsorption amount were compared. The experimental data were fitted with the Langmuir model and analyzed with a pseudo-second-order kinetic process.

## 2. Materials and Methods

### 2.1. Chemicals

**Scheme 1:**
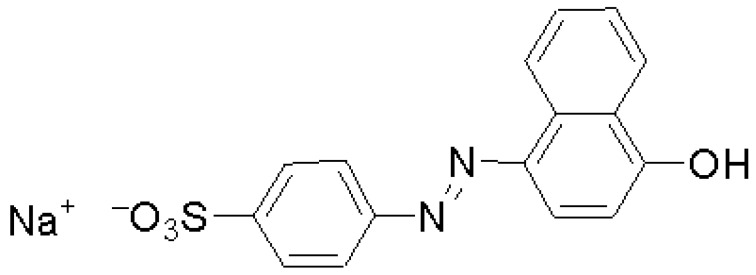
The chemical structure of Orange I.

Bent (99%, 500 mesh, Shanghai Aladdin Bio-Chem Technology Co. Ltd., Shanghai, China) should be acid-activated by 0.5 M H_2_SO_4_ at 60 °C for 24 h before use. The acid-activated process is the most widely-used technique for bentonite treatment before its further usage. After the acid-activation, the bentonite can be modified easily, and its adsorption ability can be improved significantly [41]. Orange 1, with a purity of 99%, was obtained from Shanghai Aladdin Bio-Chem Technology Co. Ltd., Shanghai China. Scheme 1 exhibits the typical chemical structure for Orange 1. Tetrahydrofuran (THF, 99%, Sinopharm, Shanghai, China) and triethylamine (TEA, 99%, Sinopharm) were dehydrated by sodium and then distillated. During experiments, several other chemicals were also used, which were utilized as received. All the chemicals such as Copper(I) bromide (CuBr; 99% purity), (3-Aminopropyl) triethoxysilane (APTES; 99% purity), 2-(dimethylamino) ethyl methacrylate (DMAEMA, 97% purity), 2-bromoisobutyryl bromide (BIBB, 98% purity) and N,N,N,N,N-pentamethyldiethylenetriamine (PMDETA, 99% purity) were obtained from Aladdin chemical company (Shanghai, China), and used as received without any further purification.

### 2.2. Preparation of Aminosilane Functionalized Bentonite (Bent-APTES)

3.0 g Bent was dispersed in a 200 mL mixed solution of ethanol and water (v/v = 75/25). 3.0 g APTES was added, stirring for 8 h at 80 °C, followed by filtering. It was then washed with water and ethanol. Lastly, the Bent-APTES solid was obtained by drying it at 60 °C for 4 h.

### 2.3. Preparation of Bent-Br

2.0 g Bent-APTES was dispersed in 50 mL THF under stirring conditions for 2 h. The solution of 2 mL BIBB in 20 mL THF was dropped into the suspension at 0 °C, with stirring for 24 h. Bent-Br solid was obtained by filtration. It was then washed several times with THF, deionized water and acetone, respectively. Lastly, it was dried overnight.

### 2.4. Preparation of Bent-PDMAEMA

1.2 g Bent-Br and a 13 mL mixture of DMAEMA and PMDETA were added to 10 mL methanol/H_2_O (v/v = 1:2). After being degassed, CuBr was added. The polymerization reaction was vigorously stirred at room temperature for 12 h, and consequently stopped by opening to the air. The as-prepared Bent-PDMAEMA was washed with water and acetone sequentially, and dried overnight.

### 2.5. Characterizations

Fourier transform infrared spectroscopy (FTIR) spectra were collected by VECTOR-22 IR spectrometer (Bruker, Rheinstetten, Germany) over the spectral range of 400–4000 cm^−1^. X-ray diffraction (XRD) patterns were recorded by D/max 2500 X-ray diffractometer (Rigaku, Tokyo, Japan) at 40 kV. The 2θ angle varied from 2° to 8°. Scanning electron microscopy (SEM) images were obtained using Quanta 200 (Philips-FEI, Eindhoven, the Netherlands). Thermal gravimetric analysis (TGA) was operated on a Diamond TG/DTA Instrument (Perkin–Elmer, Norwalk, CT, USA) under a nitrogen flow in the temperature ranges of 25 to 800 °C at a heating rate of 10 °C·min^−1^. UV-vis spectra were obtained on TU-1901 UV/VIS Spectrophotometer (Purkinje General, Beijing, China) at an optimal wavelength of 476 nm.

### 2.6. Batch Adsorption Experiments

0.02 g Bent-PDMAEMA was added into 40 mL Orange I solution by agitation for 3 h. The adsorption amount of Bent-PDMAEMA for Orange I was calculated using the following equation:

(1) $$\;q_{e}=\frac{C_{0}-C_{e}}{M}\times V$$

where *q*_e_ is the adsorption amount of Orange I per unit weight of adsorbent (mg·g^−1^), *c*_0_ is the initial Orange I concentration (mg·L^−1^), *c*_e_ is the equilibrium concentration of solute in the bulk solution (mg·L^−1^), *M* is the mass of Bent-PDMAEMA (g) and *V* is the solution volume (L).

### 2.7. Adsorption Isotherms

#### 2.7.1. Langmuir Isotherm

If there is no interaction between adsorbed molecules and the adsorbed molecules arranged on the interface are monolayer, it follows the Langmuir model, which can be expressed as follows:

(2) $$\frac{c_{e}}{q_{e}}=\frac{1}{k_{L}q_{0}}+\frac{c_{e}}{q_{0}}$$

where *q*_0_ is the maximum adsorption amount (mg·g^−1^) and *k*_L_ is the Langmuir adsorption constant.

#### 2.7.2. Freundlich Isotherm

The heterogeneous adsorption on a surface might follow the Freundlich model, which can be expressed as follows:

(3) $$\;\ln q_{e}=b_{F}\ln c_{e}+\ln k_{F}$$

where *b*_F_ is an adsorption constant, and *k*_F_ is the Freundlich constant.

### 2.8. Adsorption Kinetics

The pseudo-first-order kinetic model was expressed as follows:

(4) $$\;\ln\left(q_{e}-q_{t}\right)=\ln q_{e}-k_{1}t$$

where *q*_t_ (mg·g^−1^) is the adsorption amount of Orange I at *t* time (min), and *k*_1_ (min^−1^) is the equilibrium rate constant of the pseudo-first-order kinetic model.

The pseudo-second order process was expressed as follows:

(5) $$\frac{t}{q_{t}}=\frac{1}{k_{2}q_{e}^{2}}+\frac{t}{q_{e}}$$

where *k*_2_ is the equilibrium rate constant of the pseudo-second-order model.

## 3. Results and Discussion

### 3.1. Characterizations of Bent-PDMAEMA

**Figure 1:**
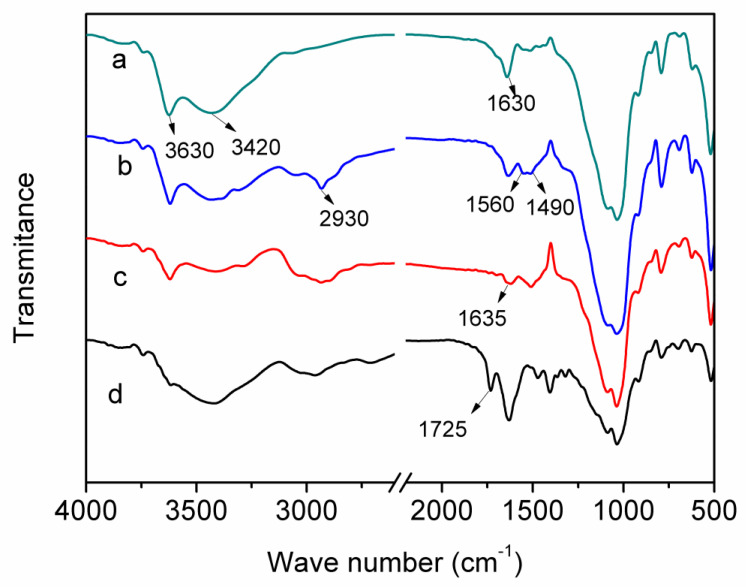
Fourier transform infrared spectroscopy (FTIR) spectra of (a) Bent, (b) Bent-(3-Aminopropyl) triethoxysilane (APTES), (c) Bent-Br and (d) Bent-poly(2-(dimethylamino) ethyl methacrylate (PDMAEMA).

FTIR spectra of the as-prepared products in every synthesis process are shown in Figure 1. The wide peaks at 3630 and 3420 cm^−1^ were hydroxyl stretch vibrations of water intercalated in layers of Bent. In addition, the peak appearing at 1630 cm^−1^ is related to the hydroxyl stretch vibration of Bent, which confirmed the existence of water molecules in the lattices of Bent [42]. After being modified with aminosilane, new peaks appeared in Figure 1b. The peak at 2930 cm^−1^ was due to –CH_2_ group stretch vibration of aminosilane. A peak at 1560 cm^−1^ was bending vibration of –NH_2_. After initiator functionalization, there was no obvious difference in Figure 1c. The signals were contributed predominantly by Bent-APTES, while the amount of initiator anchored to Bent-APTES was too small to be observed. This phenomenon was consistent with the published work. After being grafted with PDMAEMA, the new band at 1725 cm^−1^ appeared in Figure 1d, reflecting C=O stretch vibration.

**Figure 2:**
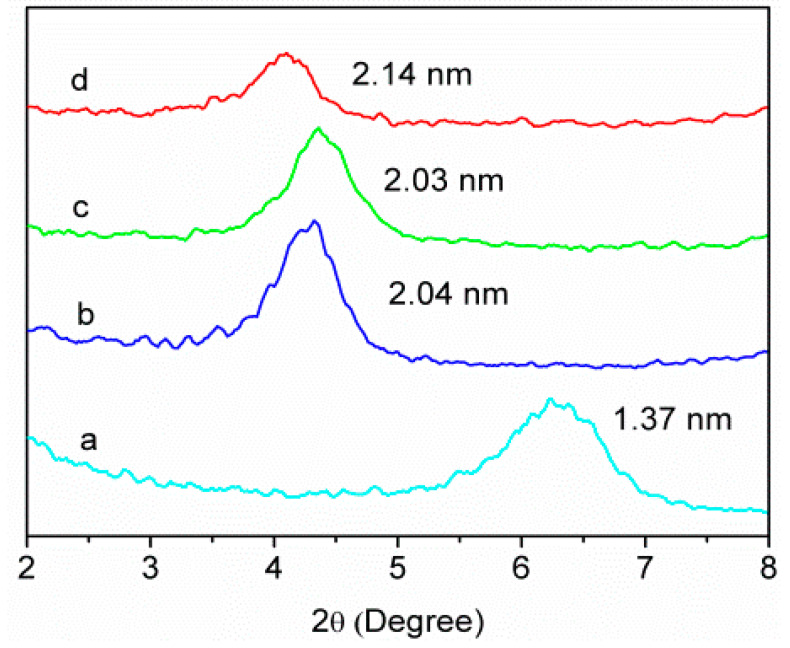
X-ray diffraction (XRD) patterns of (a) Bent, (b) Bent-APTES, (c) Bent-Br and (d) Bent-PDMAEMA.

Figure 2 shows XRD patterns of all the prepared materials. A diffraction peak appeared at 2*θ* = 6.48°, showing the (001) plane of Bent. The basal spacing for Bent was calculated to be 1.37 nm. Compared with the natural Bent, there were obvious changes in the peak position for Bent-based modified materials, indicating that the interlayer space of Bent was intercalated by organic groups. As for Bent-APTES, Bent-Br and Bent-PDMAEMA, basal spacing increased to 2.04, 2.03 and 2.14 nm, respectively.

**Figure 3:**
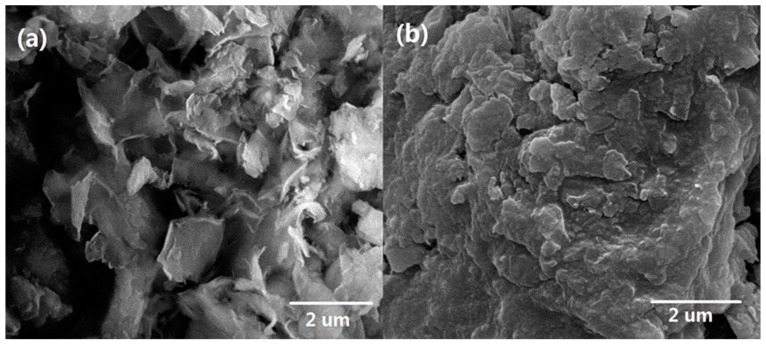
Scanning electron microscopy (SEM) images of (**a**) Bent and (**b**) Bent-PDMAEMA.

The polymer brushed grafted on the Bent can change the image of the material. Figure 3 shows compared SEM images of the modified Bent. The characteristic schistose structure with an irregular fold of clay can be seen in Figure 3a. For comparison, the surface of Bent-PDMAEMA is shown in Figure 3b. Meanwhile, the smooth and compact structure caused by the coverage of polymers appeared, proving the successful modification of polymer brushes on Bent.

**Figure 4:**
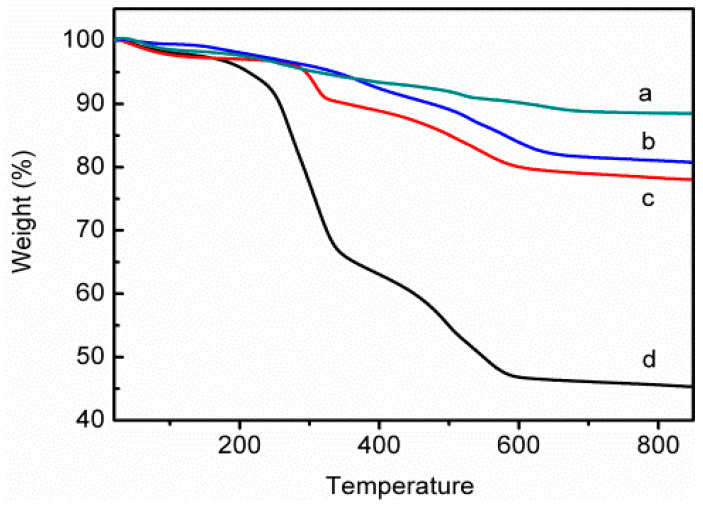
Thermal gravimetric analysis (TGA) analysis of (a) Bent, (b) Bent-APTES, (c) Bent-Br and (d) Bent-PDMAEMA.

The thermostability of the materials are very important in practical applications. Here, Figure 4 shows the results of TGA measurement of Bent-PDMAEMA and its precursors. It can be seen from Figure 4a that the weight loss of Bent was 6%, which was caused by the desorption of water. The weight loss due to the desorption of water in Bent was due to two different aspects, i.e. in the first phase, the desorption was due to the adsorbed water available on the intercalated layers of the Bent, while in the other phase the desorption was due to the release of water or OH^−^ existing in the lattices of Bent [42]. Therefore, the chemidesorption of adsorbed water available on the intercalated layers of Bent was evolved at around 200–350 °C, while the release of water or OH^−^ from the lattices of Bent was at around 700 °C. When modified with organic groups, more weight loss of Bent-APTES appeared due to aminosilane (Figure 4b,c). After the initiator was anchored, the weight loss of Bent-Br was not obvious, due to the small amount of initiator. The most weight loss of 33.2 wt.% appeared in Figure 4d, which was caused by the removal of PDMAEMA with large mass. This phenomenon also provides support for the modification of Bent with polymer brushes.

### 3.2. Effect of pH

**Figure 5:**
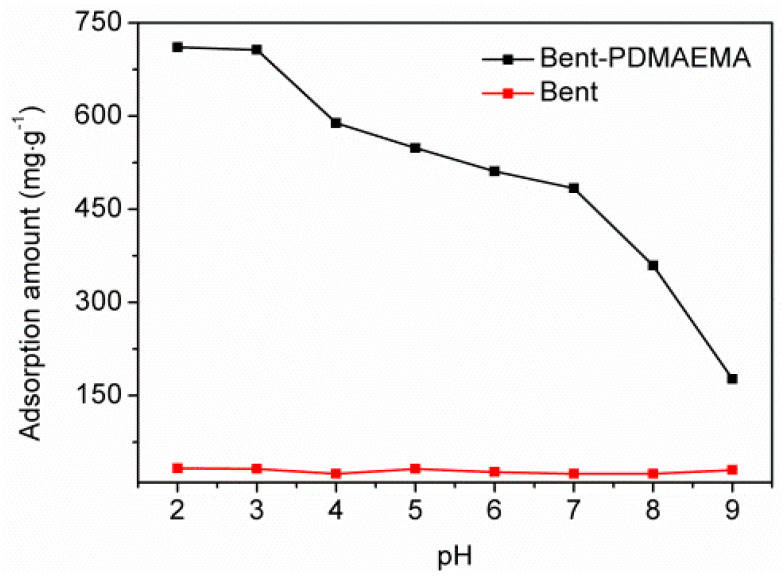
Effect of pH on the adsorption amount of Orange I on Bent and Bent-PDMAEMA.

To some extent, the properties of an adsorbent depends on the pH value of the solution. Figure 5 shows the effect of pH on the adsorption amount of Orange I at the initial Orange I concentration of 500 mg·L^−1^. As for Bent, the adsorption amount of Orange I only depended on the physical adsorption. Obviously, the adsorption amount of Bent for Orange I kept stable at 30 mg·g^−1^ in pH 2.0–9.0. Meanwhile, as for Bent-PDMAEMA, Orange I can be adsorbed through both physical interaction and electrostatic action. Orange I was adsorbed on Bent by the electrostatic adsorption between –NH(CH_3_)_2_^+^ of polymer brushes and –SO_3_^2−^ of Orange I. Namely, the mechanism of adsorption is the reduplicate action that can improve the adsorption amount of Orange I. Therefore, pH had an obvious effect on the adsorption property of Bent-PDMAEMA for Orange I. When the pH was less than 3, the adsorption amount reached 700 mg·g^−1^. When further increasing pH, the adsorption amount obviously decreased. The decrease of pH showed the increase of the hydrogen ion amount. This made the –N(CH_3_)_2_^+^ groups of PDMAEMA easily protonated into –NH(CH_3_)_2_^+^ groups. When pH increased, –NH(CH_3_)_2_^+^ groups were gradually deprotonated, which is not beneficial for the electrostatic adsorption. At the same time, the positive charges distributed on the polymer brushes made Bent-PDMAEMA disperse easily in the solution. At higher pH values, the polymer brushes were agglomerated. Under this situation, –N(CH_3_)_2_^+^ was ‘buried’ in the polymer aggregate. The real amount of interactive points decreased.

### 3.3. Effect of Initial Concentration of Orange I

**Figure 6:**
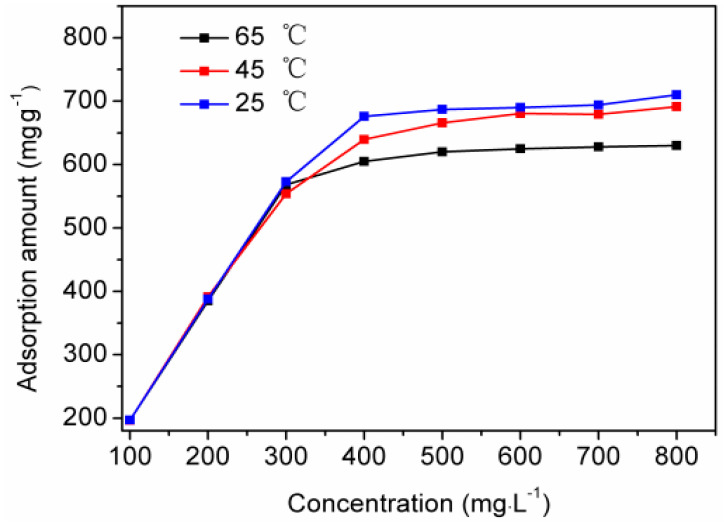
Effect of the initial concentration of Orange I solution on the adsorption amount of Orange I on Bent-PDMAEMA at 25, 45 and 65 °C.

The effect of initial Orange I concentration on adsorption amounts of 25, 45 and 65 °C was investigated, as shown in Figure 6. At a certain temperature, the adsorption amount increased with the increase of the initial Orange I concentration. In the lower concentration, the adsorption amount increased quickly with the increase of Orange I concentration. At higher concentration, the adsorption amount remained stable. At that time, the total available adsorption sites have been occupied. In addition, with the increase of the solution temperature, the adsorption amount of Bent-PDMAEM for Orange I decreased, indicating that the adsorption process was an exothermic process.

**Figure 7:**
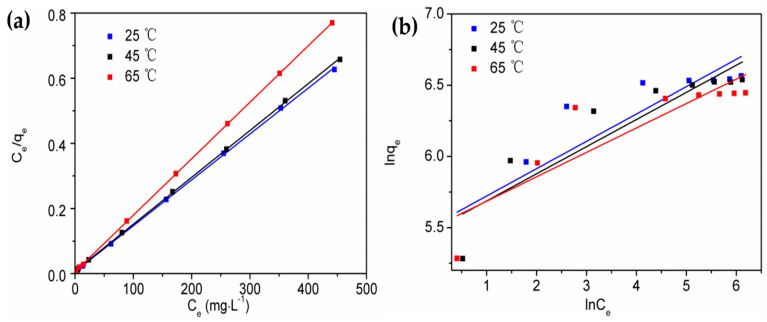
The fitting curves of the experimental data by the (**a**) Langmuir and (**b**) Freundlich models.

In Figure 7, the adsorption isotherms have been deduced using the Langmuir and Freundlich models, respectively, at different temperatures. Obviously, the fitting curve according to the Langmuir model showed a correlation coefficient of 0.99—far more precise than according to the Freundlich model, indicating that the real reaction was monolayer adsorption.

### 3.4. Effect of the Contact Time

**Figure 8:**
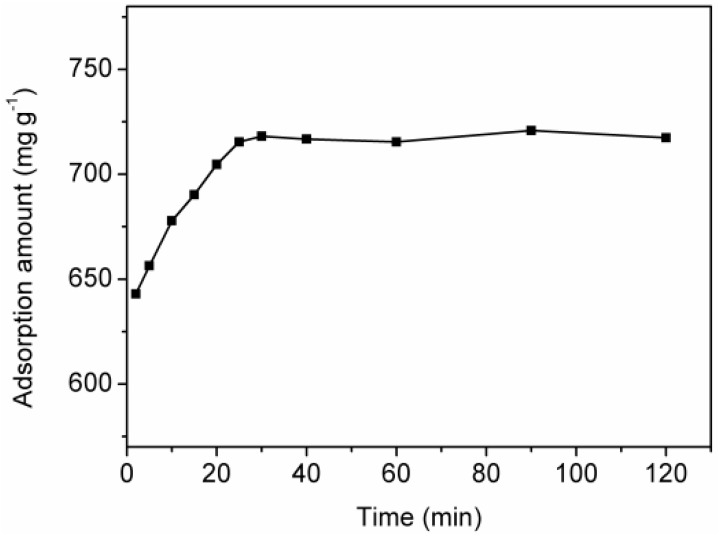
Effect of the contact time on the adsorption amount of Orange I on Bent-PDMAEMA.

Figure 8 shows the effect of the contact time on adsorption property. The adsorption rate was fast in the initial process, and a relatively slower adsorption rate was then observed. The rapid adsorption process occurred in the first 30 min. After that, the adsorption sites were occupied, making the adsorption rate slow down, and reach the equilibrium state. The fast adsorption during the initial period made the adsorbent convenient in usage.

**Figure 9:**
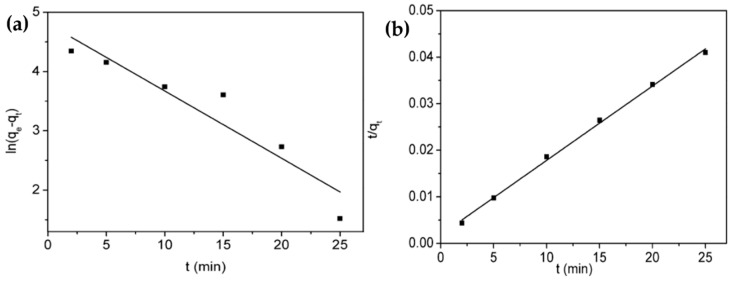
Plots of pseudo-first-order (**a**) and pseudo-second-order (**b**) kinetic models.

In Figure 9, the adsorption kinetics curves were fitted with pseudo-first-order and pseudo-second-order models, respectively. Compared with the curve in Figure 9a, the curve in Figure 9b showed a better fitting result, indicating that the experimental data fitted well with a pseudo-second-order model. The correlation coefficients of the pseudo-second-order model were higher than 0.99.

### 3.5. Comparison of Adsorption Ability of Bent-PDMAEMA with other Materials

Due to its specific morphology, the prepared Bent-PDMAEMA material exhibited excellent adsorption abilities, with a maximum adsorption amount of ~700 mg·L^−1^. The observed adsorption of orange dye using Bent-PDMAEMA material is higher than other adsorbents such as Fe_3_O_4_-CS [43], Iron-benzenetricarboxylate [44], MgAl-layered double hydroxides [45] and cross-linked porous polyimide [46] towards other orange dyes. Table 1 exhibits a comparison for the adsorption ability of Bent-PDMAEMA with other adsorbent materials towards various orange dyes.

## 4. Conclusions

In summary, a new kind of adsorbent was prepared and marked as Bent-PDMAEMA by the SI-ATRP method. The structure and properties of the modified Bent with polymer brushes were verified by FTIR, XRD, SEM and TGA analysis. All the results proved the successful preparation of Bent-PDMAEMA. Compared with the initial Bent, the adsorption ability of Bent-PDMAEMA for Orange I was improved significantly. By detailed experiments, however, it was observed that the adsorption process was affected by several process parameters, such as the pH value of Orange I solution, the initial concentration of Orange I and the contact time. The prepared Bent-PDMAEMA material exhibited a maximum adsorption of ~700 mg·L^−1^ at pH 3.

## Figures and Tables

**Table 1:** Comparison of the adsorption ability of different adsorbents towards various Orange dyes.

Adsorbent Materials	Maximum Dye Adsorption	Reference
Fe_3_O_4_-CS for Orange I	183.2 mg·g^−1^	[43]
Iron-benzenetricarboxylate for Orange II	435 mg·g^−1^	[44]
MgAl-layered double hydroxides for acid Orange 10	1.47 mmol·g^−^^1^	[45]
Cross-linked porous polyimide for Orange 10	500 mg/L	[46]
Bent-PDMAEMA for Orange I	700 mg·L^−1^	Present work
